# The Interactions of Oxygen with Small Gold Clusters on Nitrogen-Doped Graphene

**DOI:** 10.3390/molecules18033279

**Published:** 2013-03-13

**Authors:** Xin Chen, Shaorui Sun, Fan Li, Xiayan Wang, Dingguo Xia

**Affiliations:** 1 College of Environmental and Energy Engineering, Beijing University of Technology, Beijing 100124, China; 2 College of Engineering, Peking University, Beijing 100871, China

**Keywords:** gold cluster, graphene, oxygen adsorption, density functional theory

## Abstract

By means of density functional theory, the adsorption properties of O_2_ molecule on both isolated and N-graphene supported gold clusters have been studied. The N-graphene is modeled by a C_65_NH_22_ cluster of finite size. The results indicate that the catalytic activity and the O_2_ adsorption energies of odd-numbered Au clusters are larger than those of adjacent even-numbered ones. The O_2_ molecule is in favor of bonding to the bridge sites of odd-numbered Au clusters, whereas for odd-numbered ones, the end-on adsorption mode is favored. The perpendicular adsorption orientation on N-graphene is preferred than the parallel one for Au_2_, Au_3_ and Au_4_ clusters, while for Au_5_, Au_6_ and Au_7_, the parallel ones are favored. When O_2_ is adsorbed on N-graphene supported Au clusters, the adsorption energies are largely increased compared with those on gas-phase ones. The increased adsorption energies would significantly facilitate the electron transfer from Au d-orbital to π* orbital of O_2_, which would further weakening the O–O bond and therefore enhancing the catalytic activity. The carbon atoms on N-graphene could anchor the clusters, which could make them more difficult to structural distortion, therefore enhance their stability.

## 1. Introduction

As a new kind of catalyst to catalyze CO oxidation reactions at low temperatures, nanosized gold clusters have recently attracted considerable interest from both the industrial and academic communities due to their unique physical and chemical properties [1,2,3,4,5,6]. It is well known that gold in its bulk form has little or no catalytic activity. However, small gold clusters exhibit drastically different fundamental properties, which may be exploited in a variety of applications such as catalysis, chemical- and bio-detectors, advanced drug delivery systems, enhanced computing systems and optoelectronics [7]. As one of the key factors to understand the catalytic mechanisms, the adsorption behavior of atomic and molecular oxygen on gold clusters has been studied. It was found that the adsorption behavior of oxygen molecules on a gold cluster strongly depends on the charge status and cluster size [8,9,10]. Furthermore, an even-odd oscillation behavior of the oxygen adsorption was found in anionic Au clusters. For neutral Au clusters, the systematic studies were deficient, and there is no consistent view on the adsorption behavior of O_2_. For instance, some studies confirmed there is no adsorption for molecular oxygen on neutral Au clusters [11], but many theoretical studies suggested that the adsorption should happen [12,13,14,15,16].

Very recently the stability or the catalytic properties of Au nanoclusters supported on graphene has attracted much attention. Graphene is a single atomic layer of hexagonal sp^2^-bonded graphite with unique zero-gap electronic structure and massless Dirac fermion behavior [17,18,19,20]. The unusual electronic and structural properties make graphene a good candidate material for the generation of faster and smaller electronic devices. Its current applications in these fields may be extended to the field of heterogeneous catalysis, as support for metal nanoparticles. An enhanced reactivity for methanol oxidation has been recently reported for small platinum clusters and palladium nanoparticles supported on graphene oxide sheets [21,22,23]. Chen *et al*.’s calculation indicates that the catalytic properties for CO oxidation are improved based on Au_16_ cluster supported on graphene [24]. However, a systematic theoretical study about the interactions of oxygen molecule with Au clusters supported on graphene is lacking. 

We report here a density functional theory (DFT)-based investigation of the interactions of oxygen molecule with small Au clusters on a nitrogen-doped graphene surface. We firstly calculated all the possible adsorption conformations of O_2_ on isolated Au*_n_* clusters (*n* = 2–10), and then the interactions of O_2_ on Au*_n_* (*n* = 2–7) clusters supported on N-doped graphene (N-graphene) were fully studied. The results obtained indicate that N-graphene is able to stabilize small Au clusters, and enhance their catalytic activity simultaneity.

## 2. Methodology

All the calculations have been performed with the Amsterdam Density Functional (ADF, version 2009.01) program package [25,26,27], which is based on the DFT of electronic structure. The Perdew–Wang parameterized (PW91) form of the generalized gradient approximation (GGA) for the exchange-correlation functional is adopted in the calculations. The gold atoms were calculated with a triple-ζ polarized (TZP) slater-type basis set, and other atoms with double-ζ polarized (DZP) set. The inner core orbitals, 1s for C, N and O, (1s–4f) for Au were kept frozen. Gold being a heavy atom, relativistic effects become important. So the scalar relativistic effects were taken into account in the present work. The N-containing graphene (C_65_NH_22_) was built which contains pyridine species. Carbon atoms on the edge of the graphene are terminated by hydrogen atoms. For all stationary states, spin multiplicity was allowed to relax: possible geometries with varying spin states were carefully checked and the ground state is determined as the one with the lowest electronic energy. What’s more, the atom charges were obtained by Multipole Derived Charge analysis (MDC-q) [28], which gives charges that reproduce by construction both the atomic and molecular multipoles.

The choice of the initial geometry is important to obtain the lowest energy structures. In the current study, we obtained the most stable structures by the following approaches: first, considering previous studies on the configurations of pure Au clusters [29,30,31], we restudied the structural properties of the neutral Au*_n_* (*n* = 2–10) clusters before investigating the interaction of Au clusters with O_2_. On the basis of the optimized equilibrium geometries of pure Au clusters, we obtained the initial structures of Au*_n_*O_2_ clusters by bonding O_2_ molecule directly on each possible nonequivalent site of the Au*_n_* clusters. For Au*_n_* clusters supported on N-doped graphene (C_65_NH_22_), we firstly relaxed the planar Au clusters for two different orientations (both parallel and perpendicular) relative to the surface, and then made O_2_ adsorbed on the complexes as explained above. All these initial structures are fully optimized by relaxing the atomic positions until the force acting on each atom is negligible and by minimizing the total energy.

An important reference point for this calculation is the adsorption energy for O_2_ adsorbed on isolated Au*_n_* clusters, as well as on N-graphene (C_65_NH_22_) supported ones. In this paper, we used the following definitions for adsorption energy. When O_2_ is adsorbed on isolated Au*_n_* clusters, the adsorption energy is calculated as:

*E*_1_ = *E*(system) – *E*(Au*_n_* clusters) – *E*(O_2_) (1)

When O_2_ is adsorbed on N-graphene supported Au*_n_* clusters, the adsorption energy is calculated as:

*E*_2_ = *E*(system) – *E*(Au*_n_*/C_65_NH_22_) – *E*(O_2_) (2)

Similarly, when Au*_n_* clusters are adsorbed on N-graphene, the adsorption energy is:

*E*_3_ = *E*(system) – *E*(C_65_NH_22_) – *E*(Au*_n_* clusters) (3)

## 3. Results and Discussion

### 3.1. The Structural and Electronic Properties of Gold Clusters

**Figure 1:**
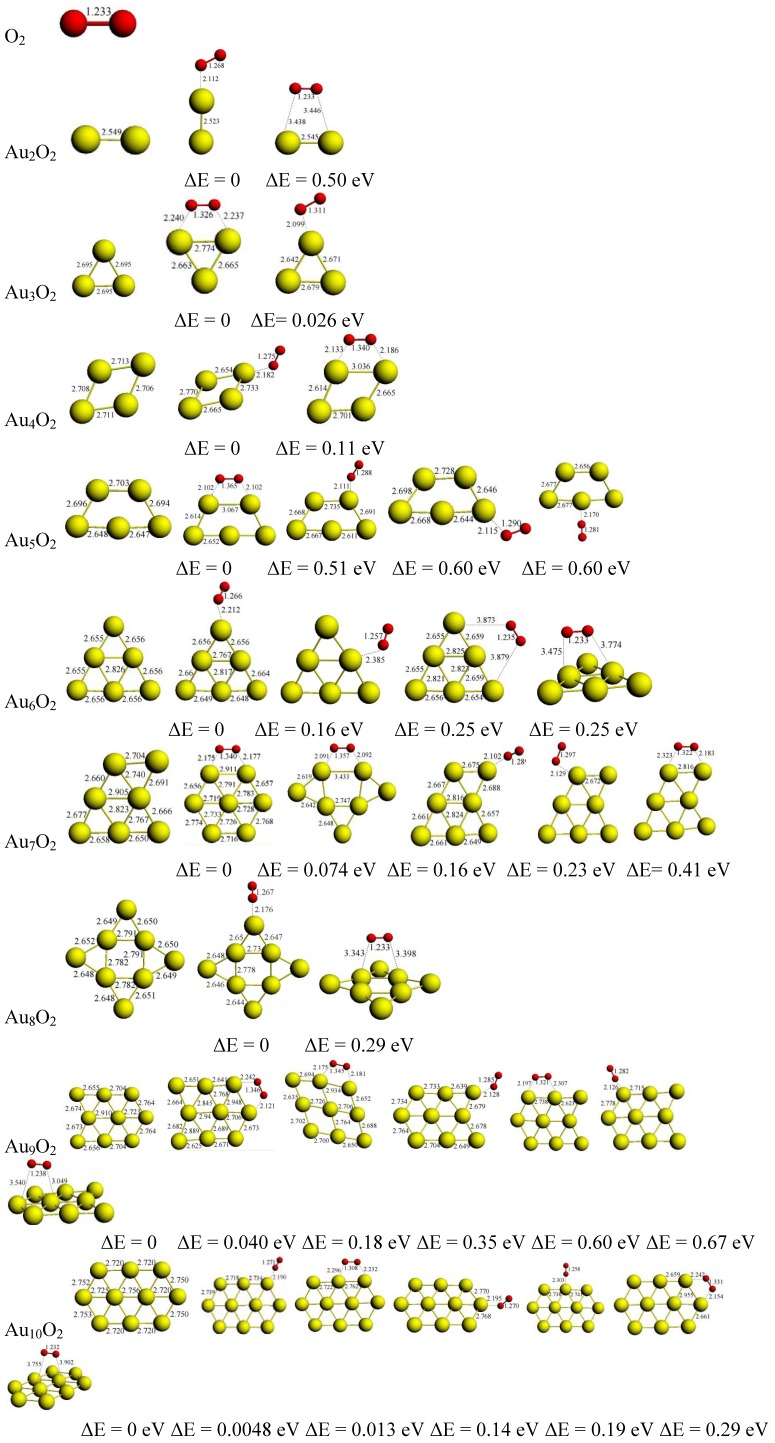
Optimized geometries for pure Au*_n_* (*n* = 2–10) clusters, single O_2_ molecule and Au*_n_*O_2_ (*n* = 2–10) complexes (distances are in angstrom).

In order to obtain the initial geometries of Au*_n_*O_2_ clusters, we first optimized isolated Au*_n_* clusters and single O_2_ molecules. The lowest energy geometries and the electronic properties of Au*_n_* (*n* = 2–10) clusters shown in Figure 1 are in good agreement with previous works [31,32,33,34,35]. The spin multiplicity, average Au–Au bond length, binding energy per atom, and the energy gap between the highest occupied molecular orbital (HOMO) and lowest unoccupied molecular orbital (LUMO) are listed in Table 1. The average Au–Au bond length and binding energy per atom increase monotonically as a function of the size of the cluster. The values of HOMO–LUMO energy gap clearly indicate an even-odd oscillation behavior in Au*_n_* clusters, that is, the even-numbered clusters have higher HOMO–LUMO gap than the odd-numbered neighbors.

**Table 1:** Calculated structural parameters of Au*_n_* (*n* = 2–10) clusters. The values in the parentheses are taken from other works.

Au*_n_* cluster	Spin multiplicity	Average bond length (Å)	Binding energy per atom (eV)	HOMO–LUMO energy gap (eV)
Au_2_	1	2.549 (2.53, 2.47) ^a,b^	1.12	2.01 (1.96) ^d^
Au_3_	2	2.695 (2.60) ^c^	1.13	1.83 (2.70) ^e^
Au_4_	1	2.710 (2.68) ^a^	1.48	0.97 (0.927) ^a^
Au_5_	2	2.678 (2.63) ^c^	1.62	0.96 (1.142) ^a^
Au_6_	1	2.712 (2.68) ^a^	1.85	2.10 (2.05) ^d^
Au_7_	2	2.722 (2.70) ^c^	1.81	1.00 (1.077) ^a^
Au_8_	1	2.695 (2.67) ^a^	1.93	1.46 (1.420) ^a^
Au_9_	2	2.739 (2.72) ^c^	1.91	0.71 (0.97) ^c^
Au_10_	1	2.742 (2.71) ^a^	1.99	1.31 (1.172) ^a^

^a^ Ref. [31]. ^b^ Ref. [36]. ^c^ Ref. [37]. ^d^ Ref. [32]. ^e^ Ref. [38].

### 3.2. The Geometries, Energetics, and the Electronic Properties of Au_n_O_2_ Complexes

#### 3.2.1. Structural Evolution

The lowest energy geometries of Au*_n_*O_2_ (*n* = 2–10) clusters and some isomers that have higher energy are displayed in Figure 1. Compared with isolated Au*_n_* clusters and single O_2_ molecule, most of the Au*_n_* geometries in their lowest energy Au*_n_*O_2_ clusters and isomers are slightly distorted, but still maintain a planar structure. This situation is believed to reflect the strong scalar relativistic effect in small Au clusters mentioned in previous studies [34]. But for Au_7_O_2_ and Au_9_O_2_, the situation is quite different and interesting. From Figure 1, it can be seen that the structures of Au_7_ and Au_9_ clusters are greatly changed after O_2_ is adsorbed on their bridge sites. In all geometries of Au_7_O_2_ complex, two evolutionary structures are obtained for Au_7_ clusters. The lowest energy structure is a planar hexagon with *D*_6h_ symmetry. The other geometry could be generated by a structural rearrangement from the former. Another structural evolution is observed in Au_9_O_2_ complex. A “bi-edge-capped-hexagon” Au_9_ structure is generated with *D*_2h_ symmetry after O_2_ is adsorbed on the bridge sites. This structure can also coexist with the most stable geometry due to its lower electronic energy. The structural evolution of these two clusters is attractive because studies suggest that this phenomenon could only occur in the temperature range of 400 to 500 K [39]. It is reported that there exists a direct correlation between stability and geometrical structures of the clusters, and relatively higher symmetry clusters are more stable [40]. This is may be one of the reasons for structural evolution after O_2_ adsorption.

#### 3.2.2. O_2_ Adsorption Energies

**Figure 2:**
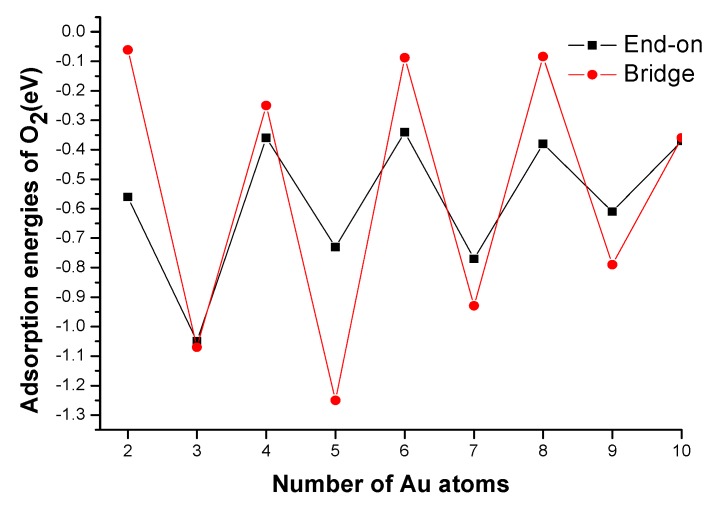
Variation of adsorption energy of molecular oxygen with cluster size.

Adsorption energy is an important index to examine the adsorption strength and the interactions between adsorbent and adsorbate. This has been investigated in some previous works for H_2_, NO, CO and H_2_O adsorption onto small Au clusters [41,42,43]. It can be seen from Figure 2 that for both end-on and bridge adsorption modes, the adsorption energies of O_2_ on odd-numbered Au*_n_* clusters are larger than those on adjacent even-numbered ones. Furthermore, for odd-numbered Au*_n_* clusters, the adsorption energies of bridge mode are also larger than those of end-on mode. On the contrary, the adsorption energies of end-on mode are larger than those of bridge mode for even-numbered ones, as shown in Figure 2. That is, the odd-numbered Au*_n_* clusters are favoring bridge adsorption of O_2_ whereas even-numbered ones are favoring end-on adsorption mode. It should be noticed that for Au_2_, Au_6_ and Au_8_ clusters, the O_2_ molecule could not adsorb on their bridge sites due to the adsorption energy is close to zero. Similarly, the O_2_ molecule could also not adsorb on the surfaces of the planar Au_6_, Au_8_ and Au_10_ clusters. The odd-even oscillation of adsorption energies for Au*_n_*O_2_ clusters is clear evidence based on the analysis above.

#### 3.2.3. Activation of O_2_ Molecules

**Figure 3:**
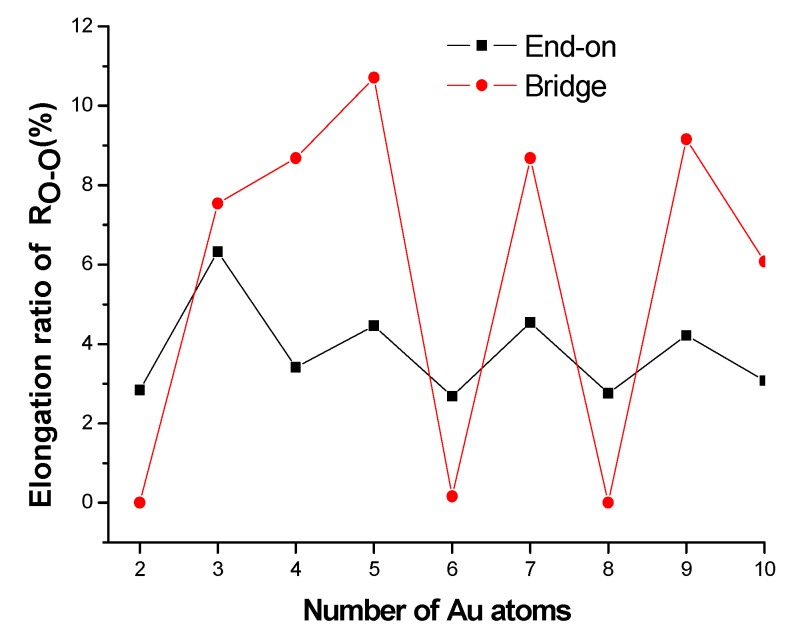
Variation of elongation ratio of R_O–O_ with cluster size.

The catalytic mechanism of oxygen reduction is to facilitate the dissociation of the O–O bond. Therefore, the catalyst’s ability to weaken the strong O–O bond and the degree of this weakening are crucial for its catalytic activity towards oxygen reduction. From Figure 3, it can be seen that all the studied Au*_n_* clusters have catalytic activity of varying degrees towards O_2_. The best catalytic activity is observed in Au_5_ cluster, which causes an ~11% O–O bond elongation. On the contrary, the Au_2_ cluster has the worst catalytic activity due to its largest HOMO–LUMO energy gap of 2.01 eV (see Table 1). Similar to the variation trends of adsorption energies, for both end-on and bridge adsorption modes, the catalytic activity of odd-numbered Au*_n_* clusters are larger than that of adjacent even-numbered ones. For odd-numbered Au*_n_* clusters, the bridge adsorption makes a larger degree of O–O bond elongation than that of end-on mode. On the contrary, a larger degree of O–O bond elongation of end-on mode is observed in the adjacent even-numbered Au*_n_* clusters.

### 3.3. The Geometries, Energetics, and the Electronic Properties of AunO_2_/N-Graphene

#### 3.3.1. Pure Au_n_ Clusters on N-Graphene

In order to analyze the interactions of oxygen with Au*_n_* clusters on N-graphene, the adsorption properties of pure Au*_n_* (*n* = 2–7) clusters supported on N-graphene were firstly considered. Both the perpendicular (┴) and the parallel (‖) orientations of the molecular axis are studied and the results are shown in Table 2. The adsorption energies (*E*_3_) obtained with Equation (3) indicate that the perpendicular orientation is preferred than the parallel one for Au_2_, Au_3_ and Au_4_ clusters. However, for Au_5_, Au_6_ and Au_7_ clusters, the most stable orientation is parallel due to their larger adsorption energies. The largest adsorption energy for the studied system is observed in Au*_7_* cluster, which value is –1.15 eV. The total MDC-q charges on the most stable gold adsorption orientation are negative in all cases, suggesting the electron transfer from the support to the metal. However, for their relatively unstable adsorption isomers, the orientation of the electron transfer is reversed. Based on the data that are presented in Table 2, the adsorption strength is mainly due to the electrostatic interactions between the clusters and support. If the Au*_n_* clusters bear more charges (whether positive or negative), the adsorption strength is stronger, otherwise the adsorption strength is relatively weak.

**Table 2:** Adsorption properties of the studied Au*_n_* (*n* = 2–7) clusters on N-graphene.

Au*_n_* cluster	Spin multiplicity	*E*_3_ (eV)	MDC-q charge
Au_2_, ┴	2	–0.58	–0.103
Au_2_, ‖	2	–0.30	0.058
Au_3_, ┴	1	–0.51	–0.107
Au_3_, ‖	1	–0.30	0.030
Au_4_, ┴	2	–0.81	–0.089
Au_4_, ‖	2	–0.57	0.080
Au_5_, ┴	1	–0.67	0.002
Au_5_, ‖	1	–0.79	–0.019
Au_6_, ┴	2	–0.64	0.098
Au_6_, ‖	2	–0.74	–0.164
Au_7_, ┴	1	–0.42	0.023
Au_7_, ‖	1	–1.15	–0.171

#### 3.3.2. O_2_ on N-Graphene Supported Au_n_ Clusters

**Figure 4:**
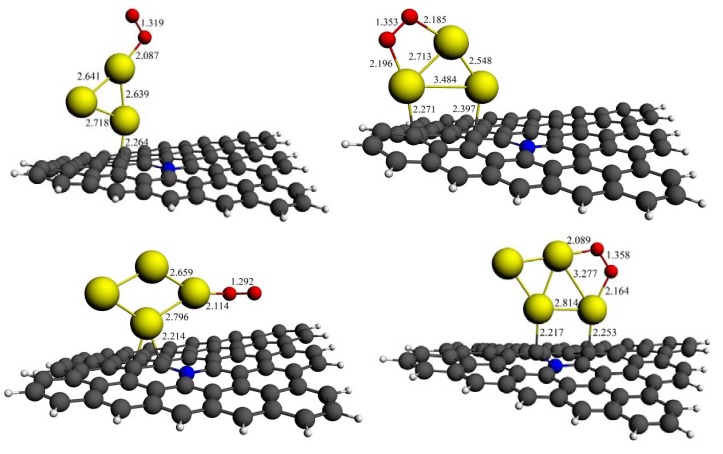
Key bond lengths for the optimized structures of the O_2_ molecule adsorbed on N-graphene supported Au_3_ and Au_4_ cluster, respectively (distances are in angstrom).

Among all the O_2_ adsorption geometries on isolated Au*_n_* clusters shown in Figure 1, we choose the most stable adsorption modes of “end-on” and “bridge” for each Au*_n_* (*n* = 2–7) cluster and then put these structures on to N-graphene’s surface. The calculated results are shown in Table 3. In all cases, the adsorption energies of O_2_ molecule on N-graphene supported Au*_n_* clusters (*E*_2_) are higher than those on isolated ones (*E*_1_) to varying degrees. There is no doubt that the increased adsorption energies would enhance the catalytic activity of small Au*_n_* clusters. For example, the O–O bond lengths on Au_3_ and Au_4_ clusters with N-graphene support is largely elongated for both end-on and bridge modes, and are longer than those on isolated ones without support. The optimized structures are shown in Figure 4. It can be seen that the Au–O bond distances in the presence of support are further shortened. At the same time, the average Au–Au bond lengths have been elongated, as shown in Figure 4. These structural changes significantly facilitate the electron transfer from Au d-orbital to π* orbital of O_2_, which could lead to a charge increasing on O_2_. From Table 3, it can be seen that there are more negative charges of O_2_ on N-graphene supported Au clusters than those on isolated ones. The calculated data indicate that the catalytic activity for oxygen reduction of Au*_n_* clusters could be improved by supporting them on N-graphene through increasing the interaction between the adsorbate and adsorbent. Actually, N-graphene itself has a good oxygen reduction activity both in acid and base solution [44,45]. Therefore, when Au clusters are supported on graphene, there may be a synergistic effect between them. This is also an important study area and needs further research.

**Table 3:** Calculated adsorption energies, *E*_1_ and *E*_2_ (eV), and net MDC-q charges, ΔQ, for O_2_ molecule in the most stable “end-on” and “bridge” adsorption with and without N-graphene support.

Au*_n_* cluster	*E*_1_ (eV)		*E*_2_ (eV)		ΔQ (O_2_)		ΔQ (O_2_, with support)	
	End-on	Bridge	End-on	Bridge	End-on	Bridge	End-on	Bridge
Au_2_	–0.56	–––	–0.83	–––	0.041	–––	–0.096	–––
Au_3_	–1.05	–1.07	–1.26	–1.69	–0.161	–0.191	–0.261	–0.272
Au_4_	–0.36	–0.25	–0.42	–0.30	–0.077	–0.073	–0.157	–0.091
Au_5_	–0.73	–1.25	–0.77	–1.32	–0.084	–0.203	–0.259	–0.223
Au_6_	–0.34	–––	–0.52	–––	–0.126	–––	–0.134	–––
Au_7_	–0.77	–0.93	–0.86	–0.99	–0.169	–0.231	–0.198	–0.248

To further clarify the enhanced catalytic activity, the adsorption energies of two important species involved in oxygen reduction, O and OH, are also calculated, as shown in Table 4. Being similar to the adsorption properties of O_2_ molecule, the adsorption energies of atomic O on N-graphene supported Au*_n_* clusters (*E*_2_) are all higher than those on isolated ones (*E*_1_). It is reported that the stronger a material binds atomic O, the more effective it will be in breaking apart molecular O_2_, which could be used to identify the efficiency of a catalyst [46,47,48], and therefore the enhanced catalytic activity is further confirmed. In addition, experimental results indicate that the strong OH adsorption on Pt may induce overpotential [49], which caused by the coverage of adsorbed OH on Pt surface and then block the adsorption of O_2_ in the next reduction step. From Table 4, it can be seen that when OH is adsorbed on N-graphene supported Au_7_ cluster, the adsorption energies are decreased. Therefore, the adsorbed Au clusters on N-graphene may also reduce the overpotential of oxygen reduction.

**Table 4:** Calculated adsorption energies of O and OH on Au*_n_* (*n* = 4, 7) clusters with and without N-graphene support.

Au*_n_* cluster	*E*_1_ (O)	*E*_2_ (O, with support)	*E*_1_ (OH)	*E*_2_ (OH, with support)
Au_4_	–3.43	–3.96	–2.75	–2.97
Au_7_, ┴	–4.21	–3.99	–3.80	–3.50
Au_7_, ‖		–4.34		–3.65

#### 3.3.3. Improved Structural Stability of Au_n_ Clusters

**Figure 5:**
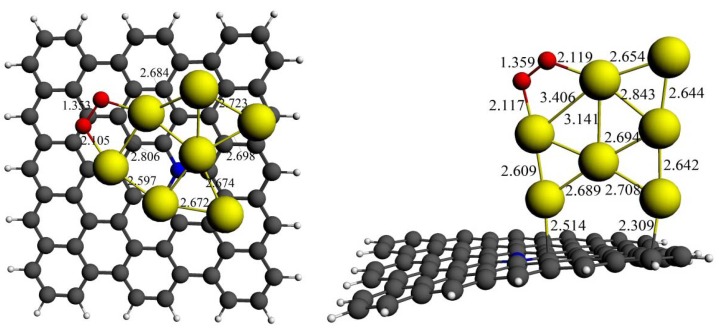
Optimized structures of the O_2_ molecule adsorbed on N-graphene supported Au_7_ cluster with bridge mode (distances are in angstrom).

As discussed in Section 3.2.1, the geometrical structures of pure Au_7_ and Au_9_ clusters would be greatly changed after O_2_ is adsorbed on their bridge sites. It is reported that the shape changes could modify the O_2_ bonding mode, therefore alter the cluster’s catalytic activity [50]. Thus, enhancing the cluster’s stability without decreasing its catalytic activity is an important issue for catalytic applications. Figure 5 shows both the parallel and perpendicular orientations of Au_7_ cluster supported on N-graphene with O_2_ adsorption. It is clearly seen that although the cluster geometry of parallel orientation has a little distortion when compared with the isolated structure, the basal cluster morphology is still maintained. The geometry of the cluster for the perpendicular case barely changed even O_2_ adsorbed on bridge sites. The reason that N-graphene enhanced the stability of Au*_n_* cluster could be attributed to the interactions between the metal atoms and the surface. For perpendicular case, the carbon atoms on N-graphene could anchor the cluster, which make it more difficult to structural distortion. In the case of parallel orientation, although there is no direct Au–C (or Au–N) interaction, the morphology of the cluster is also difficult to change due to strong adsorption energy between the cluster and the surface (–1.15 eV), as shown in Table 2.

## 4. Conclusions

By means of density functional theory, the adsorption properties of O_2_ on both isolated and N-graphene supported gold clusters have been studied. Our results indicate that the adsorption energies of O_2_ on odd-numbered Au clusters are larger than those on adjacent even-numbered ones. Similarly, the catalytic activity of odd-numbered Au*_n_* clusters, which is measured by the O–O bond weakening, is also higher than that of neighboring even-numbered ones. The odd-even oscillation of adsorption energies for Au*_n_*O_2_ is clearly evident. Furthermore, the O_2_ molecule is in favor of bonding to the bridge sites of odd-numbered Au*_n_* clusters, whereas for odd-numbered ones, the end-on adsorption mode is favored.

The adsorption energies on N-graphene of all studied clusters are in the range from –0.30 to –1.15 eV. The perpendicular orientation is preferred than the parallel ones for Au_2_, Au_3_ and Au_4_ clusters, whereas for Au_5_, Au_6_ and Au_7_, the situation is quite the contrary. Charge analysis suggests that the adsorption strength is mainly due to the electrostatic interactions between the clusters and support. 

When O_2_ is adsorbed on N-graphene supported Au*_n_* clusters, the adsorption energies are largely increased compared with those on isolated ones. The increased adsorption energies could significantly facilitate the electron transfer from Au d-orbital to π* orbital of O_2_, which could further weaken the O–O bond and therefore enhancing the catalytic activity. This is also confirmed by the increased adsorption energy of atomic O on N-graphene supported Au*_n_* (*n* = 4, 7) clusters. The carbon atoms on N-graphene could anchor the clusters and make it more difficult to structural distortion, therefore enhance their stability.
